# Pupil Dilation with Intracameral Epinephrine Hydrochloride during Phacoemulsification and Intraocular Lens Implantation

**DOI:** 10.1155/2016/4917659

**Published:** 2016-01-20

**Authors:** A-Yong Yu, Hua Guo, Qin-Mei Wang, Fang-Jun Bao, Jing-Hai Huang

**Affiliations:** The Eye Hospital of Wenzhou Medical University, Wenzhou 325000, China

## Abstract

*Objective*. To investigate mydriatic effect of intracamerally injected epinephrine hydrochloride during phacoemulsification and intraocular lens (IOL) implantation.* Methods*. Eighteen cataract patients for bilateral phacoemulsification were enrolled. To dilate pupil, one eye was randomly selected to receive intracamerally 1 mL epinephrine hydrochloride 0.001% for 1 minute after corneal incision (intracameral group), and the contralateral eye received 3 drops of compound tropicamide 0.5% and phenylephrine 0.5% at 5-minute intervals 30 minutes before surgery (topical group). Pupil diameters were measured before corneal incision, before ophthalmic viscoelastic device (OVD) injection, after OVD injection, before IOL implantation, and at the end of surgery.* Results*. At each time point, the mean pupil diameter in the intracameral group was 2.20 ± 0.08, 5.09 ± 0.20, 6.76 ± 0.19, 6.48 ± 0.18, and 5.97 ± 0.24 mm, respectively, and in the topical group it was 7.98 ± 0.15, 7.98 ± 0.15, 8.53 ± 0.14, 8.27 ± 0.16, and 7.93 ± 0.20 mm, respectively. The topical group consistently had larger mydriatic effects than the intracameral group (*P* < 0.05). The onset of mydriatic effect was rapid in the intracameral group. There was no difference in surgical performance or other parameters between groups.* Conclusions*. Intracameral epinephrine hydrochloride appears to be an alternative to the mydriatic modalities for phacoemulsification and IOL implantation. In comparison with topical mydriatics, intracameral epinephrine hydrochloride offers easier preoperative preparation, more rapid pupil dilation, and comparable surgical performance.

## 1. Introduction

Adequate mydriasis is crucial for cataract surgery, and inadequate mydriasis may be associated with increased incidence of surgical complications, including iris damage, incomplete removal of cortical and nuclear material, posterior capsule rupture, and cystoid macular edema [1, 2]. Mydriasis for cataract surgery is usually achieved preoperatively by topical mydriatics, such as cyclopentolate, tropicamide, and phenylephrine. However, low drug contact time and poor ocular bioavailability due to drainage of solution, tear turnover and its dilution, or lacrimation are the most challengeable problems associated with topical mydriatics. Long time for preparation and repeated usage of eye drops are needed to make up for the low infiltration of topical mydriatics, which is apt to cause ocular surface toxicity or other systemic complications [3, 4]. It is increasingly concerned to find an alternative to the mydriatic modalities for cataract surgery with easier preoperative preparation, more rapid pupil dilation, and comparable surgical performance compared with routine topical mydriatics.

Epinephrine is a substance with dual effects to contract the dilator musculature by its *α* receptor actions and relax the sphincter by a *β* effect, resulting in mydriasis. It is usually used at a low concentration in the irrigating solution to maintain mydriasis during cataract surgery [5, 6] and reduce the risk of iris damage in patients with intraoperative floppy iris syndrome [7]. Epinephrine has been reported to be a more potent mydriatic than phenylephrine in the porcine eyes [8]. However, epinephrine may also stimulate receptors in cardiovascular tissues, resulting in systemic side effects, such as elevation of blood pressure [9, 10]. It is still in argument whether epinephrine is a promising alternative to routing topical mydriatics for cataract surgery. This study assessed pupil dilation by intracameral injection of epinephrine hydrochloride and compared the overall surgical performance and postoperative outcomes with those of routine topical mydriatics for phacoemulsification and intraocular lens (IOL) implantation.

## 2. Patients and Methods

### 2.1. Patients

This prospective, randomized, and double blind study enrolled subjects scheduled for bilateral phacoemulsification and IOL implantation. The exclusion criteria included the following: (1) the patient with asymmetrical lens classification of the two eyes according to the Lens Opacities Classification System III who was excluded to keep lens density homogeneity in cataract between the 2 groups, (2) patients using eye drops or taking systemic medications that could affect pupil dilation, (3) ocular pathologies other than cataract, such as corneal disease, anisocoria, malformations of the anterior segment, glaucoma, uveitis, and diabetic retinopathy, and (4) a history of previous intraocular surgery or ocular trauma.

The research protocol adhered to the tenets of the Helsinki Declaration and was approved by the local ethics committee. The study was registered at https://www.clinicaltrials.gov/ (NCT01264653). All patients were fully informed about the details and possible risks inherent to this study. Written informed consent was obtained from each patient.

### 2.2. Pupil Dilation

The completely random series was created by the commercial software (SPSS, ver. 13.0; SPSS, Chicago, USA). To dilate pupil for surgery, one eye of each patient was randomly selected as the intracameral group to intracamerally receive 0.1 mL epinephrine hydrochloride 0.01% (adrenaline hydrochloride, Tianjin Jinyao Amino Acid, China) in 0.9 mL of balanced salt solution (BSS Plus, Alcon, USA), giving a concentration of 1 : 100000. The solutions were prepared at the start of surgery by the surgeon (A-Yong Yu), given intracamerally just after cornea incision, and stayed at anterior chamber for 1 minute. It took approximately 3 minutes to complete the whole procedure mentioned above. No placebo eye drops were used in the intracameral group. The contralateral eye, as the topical group, received 3 drops of topical mydriatics (Mydrin, Santen, China), comprising a mixture of tropicamide 0.5% and phenylephrine 0.5%, given topically at 5-minute intervals 30 minutes before surgery. Irrigating solution with 0.4 mL of epinephrine hydrochloride 0.01% in 500 mL balanced salt solution was used in both groups.

### 2.3. Surgical Procedure

For every patient, surgery was performed in one eye selected randomly, and surgery for the contralateral eye was performed 2 days later. For topical anesthesia, 3 drops of tetracaine 0.05% were given at 5-minute intervals before surgery. The anaesthesia was the same in all eyes. All cataract surgeries were performed by the same surgeon (A-Yong Yu) using the same three-step temporal clear corneal incision (3.0 mm). Continuous curvilinear capsulorhexis with an approximate diameter of 5.5 mm was created. The lens was removed using a quick-chop technique (Infiniti Vision System; Alcon, USA). The IOL was implanted in the capsular bag. The incision was closed by hydration without sutures. The ultrasound time and energy, total surgical time, and intraoperative complications were recorded.

### 2.4. Pupil Diameter Measurement

Pupil sizes during surgery were registered from video recordings connected to an operation microscope and measured by a masked examiner using image analysis system similar to that described by Lundberg and Behndig [8, 11]. The width of the blade of a 3.00 mm knife held at the incision site and the horizontal and vertical pupil diameters were measured in pixels directly on the same monitor screen. Thus the pupil diameter was calculated in millimeter as follows: mean pupil diameter/blade width × 3.00. To compensate for the change in magnification of the microscope, all pupil sizes referred to the pupil viewed and measured through horizontal cornea diameter magnification. The mean pupil diameter was calculated for each eye before corneal incision (T1), before the ophthalmic viscoelastic device (OVD) injection (T2), after the OVD injection (T3), before IOL implantation (T4), and at the end of surgery (T5).

### 2.5. Corneal Measurement

Preoperatively and 1 month postoperatively, the central corneal thicknesses (CCT) was measured with a Scheimpflug imaging system (Pentacam, Oculus, Germany) by a masked examiner as described previously [12, 13]. Corneal endothelial density (CED) was measured from a central cluster of 50 cells from central corneal endothelial photographs with a specular microscope (SP-2000P, Topcon, Japan) by a masked examiner.

### 2.6. Measurement of Blood Pressure and Heart Rate

Blood pressure and heart rate were assessed with an electrocardiomonitor (MP2 IntelliVue, Philips, Netherlands) on the upper arm of patients during surgery, as reported previously [14]. Data were recorded simultaneously to the pupil size measurements to assess the systemic side effects.

### 2.7. Other Measurements

The refraction and best corrected visual acuity (BCVA) were measured preoperatively and 1 day, 1 week, and 1 month postoperatively by a masked examiner. The intraocular pressure (IOP) was measured preoperatively and 2 hours, 1 day, 1 week, and 1 month postoperatively by noncontact tonometry (TX-F, Canon, Japan) by a masked examiner.

### 2.8. Statistical Analysis

Data were first collected on standardized case-report forms and then entered into a central database for analysis. Statistical analysis was performed with commercial software (SPSS, ver. 13.0; SPSS, Chicago, USA). Normality of data was checked by the Kolmogorov-Smirnov test. Descriptive statistics for continuous variables were calculated as means and standard deviations (SDs). For averaging, visual acuity was converted to logMAR value. Differences in pupil diameter, CCT, BCVA, IOP, blood pressure, and heart rate were assessed by repeated-measures analysis of variance (Re-ANOVA). Post hoc paired *t*-tests were used to determine statistically significant pairwise differences. The calculated sample size was 12 patients to offer 90% statistical power at the 5% level to detect a 0.3 mm difference in pupil diameters between the two groups, when the SD of the mean difference was 0.25 mm. The level of significance was *P* < 0.05.

## 3. Results

Thirty-six eyes of 18 patients (7 females and 11 males) were enrolled in the study, and the average age was 63.78 ± 3.10 years.

### 3.1. Mydriatic Effect

As Table 1 showed, both groups showed a significant mydriatic effect intraoperatively. The topical group consistently had a larger mydriatic effect than the intracameral group. The onset of mydriatic effect was rapid in the intracameral group, within 1 minute after intracameral injection of epinephrine. The maximum effect in both groups was seen just after the injection of OVD, with no significant change in effect toward the end of surgery.

### 3.2. Surgical Performance

The operations lasted for a mean time of 16.75 ± 0.94 minutes in the intracameral group and 13.00 ± 0.60 minutes in the topical group (*P* = 0.002). The mean phacoemulsification time was 12.51 ± 3.55 seconds in the intracameral group and 10.06 ± 2.53 seconds in the topical group (*P* = 0.571). And the percentage of effective phacoemulsification energy was 8.97 ± 1.77 and 7.47 ± 1.23, respectively (*P* = 0.491).

Iris prolapsed from the main incision in one eye in the intracameral group, and the surgery was performed successfully. No other intraoperative or postoperative complications occurred in either group.

### 3.3. Cardiovascular Effect

Significant increases in heart rate and systolic and diastolic blood pressure occurred in both groups preoperatively (Table 2), especially in systolic blood pressure. But the difference was not significant between groups (Re-ANOVA, *P* = 0.751, 0.673, and 0.993, resp.).

### 3.4. IOP

As Table 3 showed, slight increases in IOP were seen 2 hours postoperatively in both groups, but the difference was not significant between groups. The IOP did not differ significantly from before operation in both groups after 1 day postoperatively.

### 3.5. CED

The preoperative CED was 2730 ± 61 cells/mm^2^ in the intracameral group and 2658 ± 50 cells/mm^2^ in the topical group (*P* = 0.366). The postoperative CED was 2448 ± 81 cells/mm^2^ and 2483 ± 71 cells/mm^2^, respectively (*P* = 0.354). A significant corneal endothelial cell loss was seen at 1 month postoperatively in both groups (*P* = 0.003 and 0.019).

### 3.6. CCT

Before and after surgery, the mean CCT in the intracameral group was 539 ± 32 *μ*m and 544 ± 41 *μ*m, respectively, and was 541 ± 26 *μ*m and 541 ± 33 *μ*m, respectively, in the topical group. No significant difference in CCT appeared within or between groups (*P* = 0.360 and 0.744 and 0.902 and 0.819, resp.).

### 3.7. BCVA

As shown in Table 4, BCVA and spherical equivalent were not significantly different preoperatively and were improved postoperatively in both groups. No significant difference in postoperative parameters was found between the two groups.

## 4. Discussion

Due to the side effects of topical mydriatics on ocular surface, poorly postponed physiological conjunctiva absorption, and systemically high absorption through the nasolacrimal system, intracameral mydriatics, as an alternative, are becoming an issue of increasing concern in the field of intraocular surgery. This study demonstrated that intracameral injection of epinephrine hydrochloride was effective for pupil dilation during phacoemulsification and IOL implantation. The onset of mydriatic effect was within 1 minute after intracameral injection of epinephrine, and the maximum effect occurred just after the injection of OVD, which stably lasted to the end of surgery. Due to the poor absorption through the cornea, epinephrine is, therefore, not useful for topical application but is proved to have pharmacologic effects identical to those of other mydriatics when injected directly into the anterior chamber of the eye [11, 15–17]. The mydriatic effect of intracameral injection of epinephrine was weaker for many routine cases compared with the topical mydriatics based on the present data, especially because of the surgically induced traumatic miosis [1, 5]. However, overall surgical performance in the intracameral group, such as the mean phacoemulsification time and effective phacoemulsification energy, was comparable with that in the topical group, indicating that the mydriatic effect of intracameral injection of epinephrine is clinically acceptable.

Clinicians had expressed concern about elevation of blood pressure or change in IOP complicated by epinephrine [18]. In this study, the change of cardiovascular system after intracameral injection of epinephrine was not significantly different from that in the topical group. Surgically induced neurogenic hypertension should be responsible for the preoperative increase in blood pressure and heart rate in both groups. In addition, postoperative change in IOP in the intracameral group did not differ significantly from that in the topical group and recovered to baseline 1 day after surgery in both groups. These demonstrated that intracameral epinephrine is comparable with topical mydriatics in the present study in terms of systemic side effects and change in IOP.

No significant difference in CED or CCT appeared between the two groups. Jeffrey et al. [19] implied that intracameral medications may cause toxic anterior segment syndrome (TASS) or corneal endothelium dysfunction. No severe complication or severe corneal endothelial cell damage appeared when epinephrine hydrochloride with a concentration of 1 : 100000 was injected intracamerally in the present study. This is in accordance with Cakmak et al. [20]. Postoperative BCVA and spherical equivalent were significantly improved in the intracameral group, which was not significantly different from the topical group.

However, the present study revealed the increased operation time in the intracameral group and consistently larger mydriatic effect in the topical group. No study had ever showed more potent intracameral mydriatics than topical mydriatics so far. The possibility of a combined use of intracameral mydriatics was studied [21, 22]. Lundberg discussed the combined use of intracameral phenylephrine cyclopentolate and lidocaine hydrochloride and reported a weak effect of phenylephrine when combined with cyclopentolate [15]. It is plausible that a combination of intracameral mydriatics and topical mydriatics would eliminate the nursing time and improve the efficacy of intracameral mydriatics, resulting in time-efficient pupil dilation [23].

In conclusion, intracameral epinephrine hydrochloride appears to be an alternative to the mydriatic modalities for phacoemulsification and IOL implantation. In comparison with topical mydriatics, intracameral epinephrine hydrochloride offers easier preoperative preparation, more rapid pupil dilation, and comparable surgical performance. The limitation of this study is that the relatively small sample size may reduce the statistical power of the outcomes. Further studies of a larger number of subjects and longer follow-up periods are warranted.

## Figures and Tables

**Table 1:** Pupil size (mm) at different stages of surgery in the two groups (mean ± SD).

Stage	Intracameral group	Topical group	*P* value
T1	2.20 ± 0.08	7.98 ± 0.15	<0.001
T2	5.09 ± 0.20	7.98 ± 0.15	<0.001
T3	6.76 ± 0.19	8.53 ± 0.14	<0.001
T4	6.48 ± 0.18	8.27 ± 0.16	<0.001
T5	5.97 ± 0.24	7.93 ± 0.20	<0.001

**Table 2:** Blood pressure (mmHg) and heart rate (bpm) at different stages of surgery in the two groups.

Stage	Systolic blood pressure		Diastolic blood pressure		Heart rate	
	Intracameral group	Topical group	Intracameral group	Topical group	Intracameral group	Topical group
T1	128.1 ± 2.4	128.1 ± 2.4	74.1 ± 1.4	74.1 ± 1.4	73.6 ± 1.6	73.6 ± 1.6
T2	144.2 ± 4.8	144.3 ± 4.5	75.9 ± 2.2	77.0 ± 2.1	81.6 ± 2.9	79.1 ± 2.4
T3	144.0 ± 6.2	148.1 ± 4.5	76.4 ± 2.9	76.8 ± 2.8	78.4 ± 2.3	80.2 ± 2.6
T4	141.4 ± 6.0	145.0 ± 4.3	76.1 ± 2.5	77.6 ± 1.6	78.4 ± 2.2	78.4 ± 2.0
T5	140.8 ± 5.8	142.7 ± 3.8	74.9 ± 2.3	76.9 ± 1.8	77.6 ± 2.3	77.2 ± 2.4

**Table 3:** Intraocular pressure (mmHg) at different time points in the two groups.

Time point	Intracameral group	Topical group	*P* value
Before operation	13.29 ± 0.70	13.82 ± 0.64	0.580
2 hours after operation	16.56 ± 1.25	17.64 ± 1.22	0.540
1 day after operation	12.88 ± 1.04	13.92 ± 1.14	0.508
1 week after operation	12.84 ± 0.94	13.05 ± 0.82	0.870
1 month after operation	12.39 ± 0.90	12.19 ± 0.76	0.867

**Table 4:** The best corrected visual acuity (BCVA, in LogMAR) and spherical equivalent (D) in the two groups (mean ± SD).

Parameter		Intracameral group	Topical group	*P* value
BCVA	Before operation	0.73 ± 0.12	0.45 ± 0.07	0.067
	1 day after operation	0.17 ± 0.05	0.18 ± 0.06	0.855
	1 week after operation	0.13 ± 0.06	0.12 ± 0.07	0.934
	1 month after operation	0.12 ± 0.05	0.18 ± 0.09	0.616

Spherical equivalent	Before operation	−5.66 ± 2.16	−5.43 ± 1.88	0.936
	1 day after operation	−0.90 ± 0.33	−0.60 ± 0.26	0.486
	1 week after operation	−1.09 ± 0.34	−0.73 ± 0.24	0.385
	1 month after operation	−1.01 ± 0.30	−0.93 ± 0.27	0.829
